# Computational Modeling to Guide the Design of Mesalazine Nanoparticles Tailored for the Incorporation of Chitosan

**DOI:** 10.3390/polym17223053

**Published:** 2025-11-18

**Authors:** Leda Maria Gorla Robusti, Fernanda Isadora Boni, Leonardo M. B. Ferreira, Natália Noronha Ferreira, Deiver Alessandro Teixeira, Maria Palmira Daflon Gremião

**Affiliations:** 1 School of Pharmaceutical Sciences, São Paulo State University, Rodovia Araraquara—Jaú Km 1, Araraquara 14800-903, SP, Brazil; ledarobusti@gmail.com (L.M.G.R.); lmb.ferreira@unesp.br (L.M.B.F.); natalia.noronha@unesp.br (N.N.F.); 2 School of Pharmaceutical Sciences, University of São Paulo, Av. Professor Lineu Prestes, São Paulo 05508-000, SP, Brazil; fernanda.boni@usp.br; 3 Federal Institute Education, Science and Technology of Mato Grosso, IFMT, Avenida Ver. Juliano Costa Marques, s/n, Bela Vista, CEP, Cuiabá 78050-560, MT, Brazil; deiver.teixeira@ifmt.edu.br

**Keywords:** inflammatory bowel diseases, computational modeling, mesalazine nanoparticles, chitosan, mucoadhesion

## Abstract

The main objective of treatment with MSZ is to ensure that the drug reaches the colon, where it exerts its therapeutic effect. However, due to pH variation throughout the gastrointestinal tract and the risk of degradation or premature absorption, a considerable portion of the drug may not reach the colon in adequate concentrations. In this study, computational modeling was combined with experimental approaches for the design of MSZ nanoparticles (MSZ-NPs) suitable for chitosan (CS) incorporation. Quantum chemical calculations and molecular modeling revealed the importance of pH as a nucleation determinant and in the growth of the MSZ complexes. At pH~1.0, cationic clusters predominated, characterized by higher interaction energies and larger volumes/surface areas. At pH~4.0, zwitterionic clusters were stabilized, whereas at pH~6.0, anionic clusters formed the most compact assemblies, with the smallest calculated volume (4817 Å^3^) and surface area (2458 Å^2^). Consistent with the computational predictions, experimental approaches showed a progressive reduction in particle size with increasing pH. Nanoparticles prepared at pH 1.5 (F1.5), 4.0 (F4.0), and 6.0 (F6.0) showed mean diameters of 937, 556, and 146 nm, respectively, with corresponding zeta potentials (ZPs) of +8.5, −22.3, and −31.6 mV. Drug precipitation efficiency was as follows: 51.6% to F1.5, 95.1% to F4.0, and 75.5% to F6.0. F4.0 and F6.0 were selected to evaluate the effect of CS incorporation. The CS incorporation resulted in a reversal in the zeta potential in formulations prepared at pH 4.0 and 6.0. When 5% CS was added during nanoparticle formation (F4.0-5 and F6.0-5), the particles were smaller in diameter and had a lower positive ZP. F6.0-5 achieved the most favorable properties and strong mucoadhesion, evidenced by the ZP shift from +26.8 mV to −1.9 mV at a pH of 6.8. The modeling and experimental approaches guided the rational design of MSZ-NPs for CS incorporation, yielding mucoadhesive nanoparticles for colon-targeted drug delivery.

## 1. Introduction

Mesalazine (MSZ, 5-aminosalicylic acid) is a first-line therapeutic agent for the treatment of inflammatory bowel diseases, including ulcerative colitis and Crohn’s disease. While ulcerative colitis is restricted to the colonic and rectal mucosa, Crohn’s disease can affect the entire gastrointestinal tract [1,2,3].

MSZ acts locally on the intestinal epithelium, reducing inflammation, alleviating symptoms, and promoting mucosal healing. Despite its clinical importance, the transport of MSZ to the colon remains limited. High oral doses are often required to achieve therapeutic levels, which can cause gastrointestinal discomfort and systemic side effects. In addition, the drug’s pH-dependent solubility and chemical instability under physiological conditions further compromise its effectiveness [4,5,6,7]. The gastrointestinal tract presents additional challenges for colon-specific delivery, including variations in hydrodynamics, fluid composition, and mucosal barriers, which directly affect drug solubility, permeability, and retention [7,8].

To overcome these limitations, nanotechnology and polymer-based delivery systems have been developed, offering unique structural and physicochemical properties and improvements to drug bioavailability, providing site-specific delivery and reducing systemic toxicity [9,10,11,12]. Among these polymers, chitosan (CS), a deacetylated form of chitin, has attracted interest. It is a natural biopolymer with versatile functional properties such as non-toxicity, biocompatibility, and biodegradability. The cationic property of chitosan is due to the free amino groups left by the partial removal of acetyl groups of chitins. The CS shows applications in various fields such as wastewater treatment, biotechnology, agriculture, aquaculture, textiles, food processing, microbiology, and pharmaceuticals [4,5,6,7,8,9]. In addition, chitosan has attracted attention due to its mucoadhesive properties, which enhanced drug retention at intestinal mucosal surfaces and favor colon-targeted delivery [10,11,12,13].

Chitosan is particularly notable for its ability to form polyelectrolyte complexes with different polyanions. The molecular weight and degree of deacetylation of CS are two of the most important characteristics of chitosan, directly influencing its physicochemical and biological properties. The degree of deacetylation (DD) represents the percentage of N-acetyl groups removed from chitin during the deacetylation process, resulting in amine groups (-NH2) in the chitosan, and influences its solubility. With a high DD (above 50%), chitosan is soluble in diluted acidic solutions due to the protonation of the amine groups. Increasing the DD results in more protonated amine groups at acidic pH, which confers a greater positive charge on the molecule [14,15]. This positive charge is responsible for many of chitosan’s supramolecular interactions and biological activities [16,17]. Polyelectrolyte complexation has been a widely used strategy in the development of particles with tailored properties [14]. These complexes are mostly formed by cationic and anionic polyelectrolytes. The polyelectrolyte complex formed by CS with hyaluronic acid, a polyanion that exhibits specific interaction with CD44 receptors expressed on the surface of intestinal epithelial cells, and hydroxypropylmethylcellulose phthalate, also a polyanion, which added gastroresistant properties to the delivery system, demonstrated the potential applicability of this technological platform for the release and/or absorption of methotrexate in the colon for the treatment of local or systemic pathologies [15]. The design of polyelectrolyte complexes based on bevacizumab and gellan gum with a surface modified with chitosan promoted charge inversion and increased this protein association efficiency [16,17]. Lipid core nanocapsules coated with CS, hyaluronic acid and hydroxypropylmethylcellulose phthalate were designed as a potential approach for the colonic delivery of camptothecin, to modulate the mucoadhesive and permeability properties of this drug to improve local and targeted action in the colon cancer cells [18]. The association of the mesalazine nanosuspension with polyelectrolyte complexes, formed with CS, hyaluronic acid, and hydroxypropylmethylcellulose phthalate, modulated the properties of MSZ nanosuspensions, enabling the production of particles with tailored characteristics. The association efficiency of these systems was greater than that obtained with systems prepared with solubilized MSZ [19].

Supramolecular interactions are non-covalent interactions involved in the formation of various nanostructures and biological mechanisms [20,21]. These interactions are less energetic than covalent bonds and are, generally, more dynamic, allowing for easy formation and disruption. This brings a wide range of advantages and challenges to the design of systems based on the manipulation of supramolecular interactions [22]. Their dynamic nature confers interesting attributes for applications that require adjustments to complex biological environments, such as mucus. However, one of the main disadvantages of these interactions is local fluctuations in temperature, pH, and ionic strength, which makes precise control of their dynamics difficult [14].

Supramolecular interactions are driven by forces between the constituent parts of the reaction medium and/or interfaces. These surface forces exhibit cumulative behavior in supramolecular interactions. In CS-based particle systems, surface forces act at the particle/dispersion medium interface and are essential to understanding the formation and stabilization of self-assembled systems, such as CS-based particles [23]. The CS-based delivery systems rely on electrostatic interactions, which play a vital role and are widely explored for producing functional supramolecular arrangements [24,25]. Other supramolecular interactions are present in particle formation and act to stabilize the nanostructure resulting from electrostatics. The particles are structurally organized and stabilized by supramolecular interactions and surface forces and depend on physicochemical properties of the components and the preparation method [26,27].

The rational design of nanostructured carriers requires a detailed understanding of supramolecular interactions, as non-covalent interactions govern the self-assembly, stability, and dynamics of drug–polymer systems [14]. Systematic experimental variation in formulation parameters, such as pH, composition, size, and surface charge, can be labor-intensive and costly. In this context, in silico modeling offers a valuable strategy for predicting ionization states, interaction energies, and aggregation behavior, thereby guiding experimental conditions. Quantum chemical calculations and molecular simulations provide detailed insights into the formation, stability, and physicochemical properties of nanosystems, complementing laboratory experiments and supporting the rational development of more efficient drug delivery systems [28,29,30,31,32,33].

In the present study, a combination of computational and experimental approaches was employed to design MSZ nanoparticles (MSZ-NPs), which were prepared using the bottom-up precipitation method, with the objective of selecting particles with properties tailored to CS incorporation. The mucoadhesive properties of these particles were then evaluated.

## 2. Materials and Methods

### 2.1. Materials

MSZ was purchased from Sigma Aldrich^®^ (St. Louis, MO, USA); 98% sodium hydroxide and 37% hydrochloric acid were supplied by L.S. Chemicals (São Paulo, SP, Brazil). Ultrapure water was produced using a Milli-Q system (Millipore—Molsheim, France). Mucin type II was from Sigma Aldrich (St. Louis, MO, USA). The chitosan used was of low molecular weight (Mw ≈ 200 kDa) and had 90–95% deacetylation (Aldrich^®^ chemistry, St. Louis, MO, USA). Ultrapure water was produced using a Milli-Q system (Millipore—Molsheim, France).

### 2.2. Methods

#### 2.2.1. Evaluation of pH on MSZ Precipitation Behavior

A MSZ solution (0.67 mg/mL) was prepared in HCl (0.1 M) and subsequently titrated using HCl (0.5 M), NaOH (0.1 M), or NaOH (1.0 M) over a pH range of 1.0–7.0, with increments of 0.3 (±0.2) at 25 °C. Measurements were carried out using a Zetasizer Nano (DLS) (Malvern Instruments, Worcestershire, UK) coupled to an MPT-Z autotitrator (Malvern Instruments, Worcestershire, UK). For each pH value, particle formation by precipitation was evaluated, and the hydrodynamic diameter and zeta potential were determined.

#### 2.2.2. Computational Analysis

Density Functional Theory (DFT) studies the ground states of atoms and their associations/interactions among themselves and with other molecular systems [34]. Unlike wave-function-based approaches typical of ab initio calculations, DFT employs electron density as the primary variable, providing a practical computational framework through the Kohn–Sham equations [35]. These equations are similar to the Hartree–Fock equations, as they are self-consistent and iteratively adjusted to a previously computed gradient.

Computational methods also allow the use of the Molecular Mechanics (MM) approach, which has a classical nature and is preferably employed by researchers investigating large molecular systems (>100 atoms), as it enables the execution and completion of calculations at a low computational cost, producing satisfactory results comparable to experimental data. In this model, the molecular energy varies as the geometry is modified due to the resistance of bonds when they are stretched, compressed, or bent. This principle allows for the determination of bond lengths and bond angles between the atoms in the system.

MM calculations use a force field model, defined as a mathematical function with adjustable parameters, to describe the potential energy and other relevant properties of the particle system. The use of a parameterized force field provides satisfactory results when compared with quantum methods [36,37,38].

The determination of ionizable species as a function of pH values was performed based on the aqueous ionization constants (pKa) of the organic molecule Mesalazine (MSZ), which possesses three ionizable sites capable of donating or accepting protons, located in the amine, carboxylic acid, and alcohol functional groups, all directly bonded to the benzene ring. Using the MarvinSketch computational package [39], which employs physicochemical parameters, it was possible to obtain the percentage distribution of protonated MSZ species at their respective pH values [40]. For the calculations performed in MarvinSketch, the following parameters were applied: “mode = macro”, “Acid/base prefix = static”, “minimal basic pKa = −2”, “maximal basic pKa = 16”, and “Temperature = 298K” [40,41].

The interaction energies, as well as the volume and surface area occupied by molecular aggregates (clusters), were investigated through quantum and classical chemical calculations using quantum-based parameterizations.

For the optimization of the molecular structures of the ionic compounds, the Density Functional Theory (DFT/B3LYP) method was employed with the 6-31+G(d,p) basis set, as implemented in the GAUSSIAN 09W package [42,43,44,45]. This method was selected because it offers adequate accuracy for the proposed objective with relatively low computational cost [31,32,46]. All atomic and molecular coordinates were unconstrained and adjusted until the minimum energy state was reached, with an energy gradient defined by the ultra-fine mode corresponding to a value of <2.0 × 10^−5^. That is, the energy difference between successive configurations had to be below this threshold to ensure convergence.

Vibrational frequency calculations were carried out for these molecular structures, even after optimization [47,48]. No negative frequencies were observed, confirming that all structures were in their minimum energy states. Thus, the optimized structures were validated through harmonic vibrational frequencies, which presented only positive values, confirming the attainment of energy minimum in the potential energy surface.

After defining the molecular conformations of the ionic compounds at their minimum energy states, the Adsorption Locator module [49], implemented in the Materials Studio software, version 20.1, was used to determine the lowest-energy cluster, i.e., the most stable species. Based on the Universal Force Field (UFF) [50], all possible interactions between compounds were evaluated, identifying the lowest-energy spatial coordinates for each system [51].

Simulations were performed for optimized molecular structures corresponding to the studied pH values, considering clusters composed of 2, 15, 18, 20, 22, 24, 28, and 30 molecules. Each simulation consisted of 10 cycles with 100,000 calculations per cycle, totaling 1 million interactions. The cluster with the lowest interaction energy was identified as the most stable configuration. The self-consistent model employed the ultra-fine parameter, adopting convergence criteria for energy (<2.0 × 10^−5^), force (<0.001), and displacement (<1.0 × 10^−5^), ensuring the ground-state configuration.

The Adsorption Locator module was also used to identify low-energy adsorption sites on periodic and non-periodic substrates, as well as to investigate preferential adsorption in mixtures of adsorbates. The method is based on Monte Carlo searches in the conformational space of the adsorbent–adsorbate system [52], conducted under a simulated annealing scheme [53], where temperature is gradually reduced to locate the global minimum energy.

During simulations, the Metropolis Monte Carlo method was applied within the canonical ensemble, using four types of random movements—conformational, rotational, translational, and regenerative—assigned to randomly selected adsorbates [54]. The simulated annealing algorithm follows the physical principle of metallic annealing, in which controlled cooling allows the system to reach lower-energy states, avoiding trapping in local minimum.

The corresponding molecular volume was calculated from the minimum-energy state of the cluster, composed of cationic, zwitterionic, and anionic species [43]. The physicochemical properties, such as molecular volume and area, were determined using the Connolly Surface method [55], which defines the boundary between the molecular structure and the surrounding medium. This method enables a detailed analysis of the molecular surface characteristics, contributing to the understanding of adsorption processes and the stability of interactions within the studied system.

For a visualization of molecules, chemical systems, and the generation of molecular structure figures, the GaussView program, version 6, was used [56].

In this work, the interaction energy between the molecular structures of the system is considered. The value of this energy indicates the stability of the system and allows us to assess whether the interaction is strong or weak, and to evaluate the physicochemical characteristics of the system, such as the solubility of the compound in water. For this, the following equation [32] is used:

(1) $$E_{interaction}=E_{total\;complex}-E_{MSZ\;(specie;cluster)\;}-E_{chitosan}$$

Interactions between organic structures may occur through either physical or chemical mechanisms. Physical interactions are characterized by the absence of significant alterations in the molecules or systems involved. The forces governing such interactions are collectively known as Van der Waals forces, encompassing both attractive and repulsive components. In contrast, chemical interactions involve modifications in the electronic structure of the molecule, arising from processes of electron sharing or even electron transfer. In the present study, only physical interactions were observed, as the calculated interaction energies between molecular structures were below 20 kcal·mol^−1^, which is consistent with non-covalent interactions.

#### 2.2.3. Preparation and Characterization of MSZ Nanoparticles with Different pH Values

MSZ was dissolved in HCl (0.5 M) under magnetic stirring (600 rpm). After complete solubilization, ultrapure water was added to obtain a final drug concentration of 13.4 mg/mL. The precipitation of MSZ was obtained by dripping different amounts of NaOH (1.0 M) to adjust the pH to 1.5, 4.0, and 6.0. The amounts of each component are described in Table 1.

MSZ was dissolved in HCl 0.5 M using a magnetic stirrer (600 rpm). After solubilization, ultrapure water was added to reach a final concentration of 13.4 mg/mL. The pH values of each solution were adjusted with NaOH (1.0 M) to pH 1.5, 4.0, and 6.0 (Table 1). Formulations corresponding to each pH value, namely pHs of 1.5, 4.0, and 6.0, were designated F1.5, F4.0, and F6.0, respectively.

All samples were diluted in water (1:10), and the diameter, PDI, and zeta potential were evaluated. Thereafter, samples were centrifuged in an Excelsa II centrifuge (Model 20nd6 BL, FANEN, São Paulo, Brazil) for 30 min at 3600 rpm, the supernatant was collected, and the free MSZ was quantified in a Cary 60 UV-Vis spectrophotometer (Agilent, Santa Clara, CA, USA) at 300 nm, using a previously validated method.

#### 2.2.4. Incorporation of Chitosan in MSZ Nanoparticles

CS incorporation into MSZ-NPs was achieved using two methods. In the first method, 3, 6, 9, or 12% CS was added to MSZ-NPs prepared at pH 4.0 (F4.0) and 6.0 (F6.0). The CS dispersion was obtained in a 0.1 M HCl solution at a concentration of 1 mg/mL, under magnetic stirring for 24 h. The pH values of the CS dispersions were adjusted to 4.0 or 6.0 for incorporation into nanoparticles prepared at pH 4.0 (F4.0) or pH 6.0 (F6.0), respectively. These samples were designated F4.0-3, F4.0-6, F4.0-9, and F4.0-12 for the samples incorporated with 3, 6, 9, or 12% CS in F4, respectively. The same proportions of CS were incorporated into the nanoparticles prepared at pH 6.0 (F6.0). These samples were designated F6.0-0.03, F6.0-6, F6.0-9, and F6.0-12. The effect of adding 5% chitosan during the preparation process of mesalazine nanoparticles obtained by acid/base precipitation at pH 4.0 (F4.0-5) and 6.0 (F6.0-5) was evaluated. All samples were analyzed using the DLS.

#### 2.2.5. Interaction of Nanocomplexes with Mucin

From the 500 µg/mL mucin solution, dilutions of 100, 200, and 300 µg/mL were prepared in 0.1 N HCl solution (pH = 1.2) or in phosphate-buffered solution (pH = 6.8). The MSZ-NP dispersion at 12 mg/mL (300 µL) was incubated with the mucin dispersion and then left in a water bath at 37 °C/1 h. After this time, the zeta potential of the samples was determined using a DLS, and the results were compared with the samples without chitosan [15,16,17,18].

## 3. Results and Discussion

### 3.1. Understanding the Role of pH in MSZ Nanoparticle Formation Using Computational and Experimental Approaches

There are several limitations in the development and optimization of particle structures. Furthermore, structural details can be difficult to determine, and systematic variations in properties such as composition, size, and surface charge can make the process time-consuming and costly. Computational simulation can be used to complement experimental approaches, enabling the rational design of formulations with improved therapeutic efficiency. Quantum chemical calculations allow for the prediction of complex phenomena such as formation, interaction, stability, and characterization of systems [29,30,31]. In this work, experimental and computational approaches were used to select the MSZ-NP most suitable for CS incorporation.

**Figure 1:**
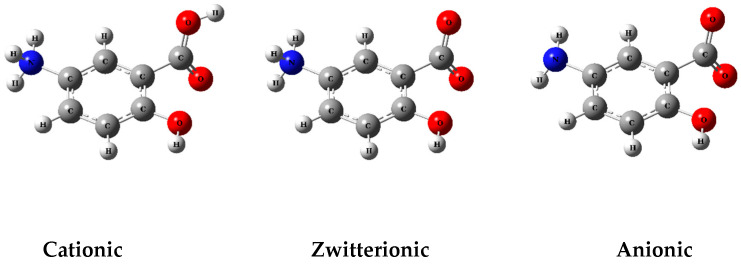
Ionizable groups of MSZ molecules in different pH conditions.

Computational analysis showed that due to the influence of pH, molecules can be ionized and remain in different ionic forms (Figure 1).

**Figure 2:**
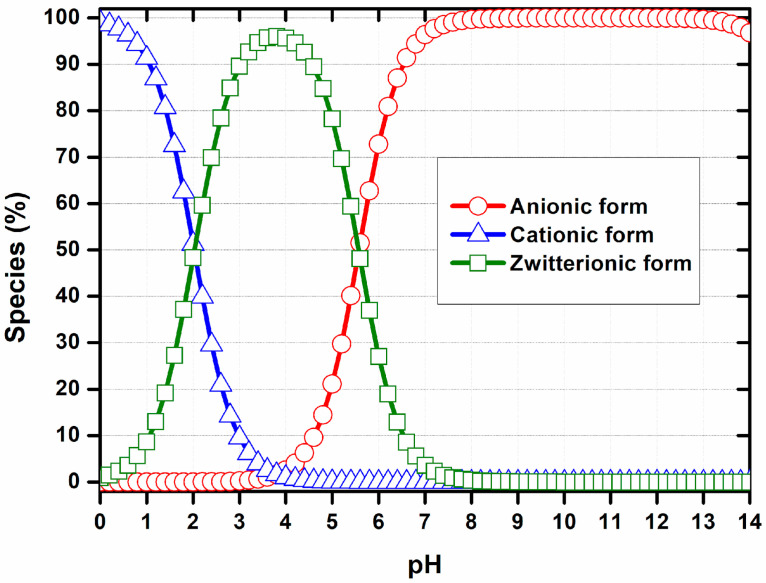
Proportion of ionic forms of MSZ according to pH. Anionic form (red), cationic form (blue), zwitterionic form (green) [39].

According to our findings, at pH values close to 1.0, MSZ molecules are predominantly in the cationic form, with the amine group fully protonated. At a pH of around 4.0, the zwitterionic form prevails, characterized by the simultaneous protonation of the amine group and ionization of the carboxyl group. At pH 6, approximately 50% of the zwitterionic species coexist with 50% of the anionic species. At a pH greater than pH 7.0, most molecules are in the anionic form, resulting from the predominant ionization of the carboxylic group (Figure 2) [39].

**Figure 3:**
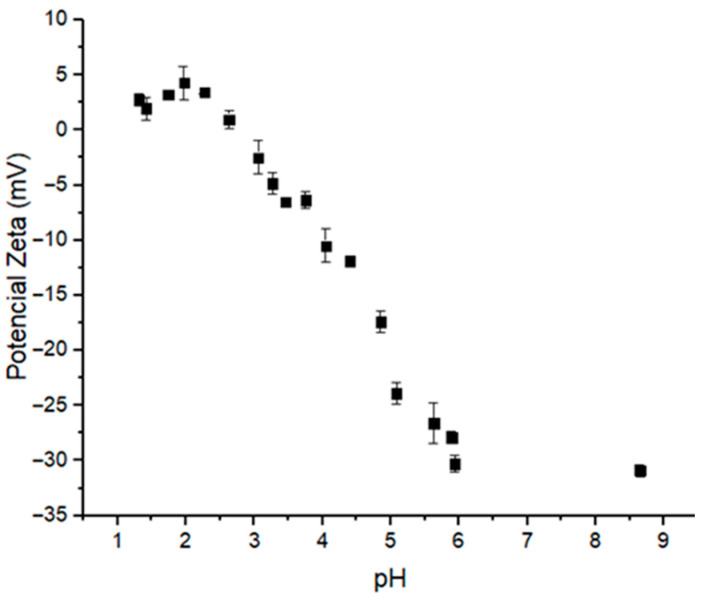
Changes in zeta potential values of the MSZ particles according to pH variation.

Numerous pharmaceuticals, such as 5-fluorouracil, methotrexate, MSZ and proteins, can influence particle formation processes involving supramolecular interactions. However, this effect is largely neglected in most studies. Therefore, selecting the pH, as well as the surface charge density, is a crucial step in the development of these systems, and measuring zeta potential is a reliable method for assessing these parameters. In this work, the influence of pH on the ionization state of MSZ was measured by titration using dynamic light scattering (Figure 3). At pH values between 1.3 and 2.6, MSZ exhibited a positive zeta potential ranging from +4.29 to +1.98 mV (Figure 3), likely due to the protonation of the amine group. This observation was consistent with computational predictions, which indicated that approximately 50% of the molecules were in cationic form at these pH values (Figure 2). At pH values above 4.5, negatively charged species predominated, with zeta potentials ranging from −11.87 to −30.9 mV (Figure 3). For pH values just below 4.5, a decrease in zeta potential was observed, suggesting a predominance of the zwitterionic form. In this configuration, MSZ molecules possess balanced positive and negative charge densities from amino and carboxylic groups, respectively, resulting in a net neutral charge.

Molecular modeling was used to understand the interaction energy between molecules of the same ionic state (Table 2), as well as the volume and area occupied by them, which are important parameters for the precipitation process (Table 3). Studying the interaction energy of chemical systems elucidates the interaction energy between molecules. Thus, the more negative the interaction energy, the stronger the interaction, indicating the minimum energy state. In this sense, the lower the energy, the more stable the complex formed [32].

Computational approaches could also be used to calculate the interaction energy of MSZ molecules. The volume and area occupied by the complexes formed under these three conditions are different (Table 3). Complexes formed by cationic molecules had higher energy interaction than the other ionic forms, regardless of the number of associated molecules, suggesting that these complexes were more stable than other forms. The anionic complexes, on the other hand, had lower interaction energy, especially after the addition of the 15th molecule, where the difference with the other ionizable forms becomes more pronounced, which may indicate a less stable complex. Increasing the number of molecules increases the interaction energy values (modulus), which suggests the formation of more stable complexes, indicating that the precipitation event is favored but needs to be controlled [29,30].

Comparing the complexes formed by 30 molecules, the anionic form obtained at pH 6.0 occupies a smaller volume (4816.85) and surface area (2457.61) than the cationic complexes formed with the same number of molecules at pH 1.5 (volume 50,008.32 and surface area 2666.82). As the number of molecules increases, the particle obtained at pH 6.0 exhibits a more compact structure, with lower volume and surface area values compared to the other forms.

**Figure 4:**
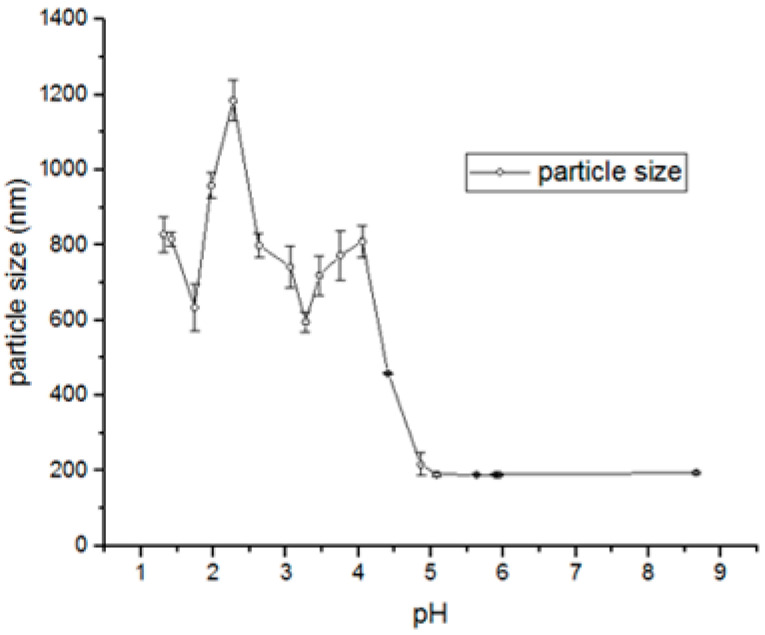
Evaluation of size (nm) of MSZ-NPs as a function of pH, using DLS.

Using the experimental approach, complexes obtained under acidic conditions (pH 1.98–3.27) displayed larger sizes, ranging from 644 to 1153 nm. At pH values above 5.0, precipitated particles were consistently around~200 nm. MSZ is highly soluble under strongly acidic (pH~1.0) or alkaline (pH > 7.0) conditions. Within the intermediate pH range, solubility decreases, leading to particle precipitation (Figure 4).

The particle sizes obtained at pH values of 6.0 and 4.0 were smaller than those obtained at a pH of 1.5. The zeta potential values of the particles obtained at a pH of 4.0 and 6.0 were negative, while at a pH of 1.5, the value was positive. The formation of mesalazine particles at these pH levels was evaluated by the percentage of drug precipitation. At a pH of 1.5, the percentage of drug precipitation was 51.6%. When increasing the pH to 4.0 and 6.0, the percentages of precipitation were much higher: 95.1% and 75.5%, respectively. The PDI values were similar (Table 4). These results demonstrate that pH strongly influences the MSZ precipitation process.

The process used to form the MSZ nanocomplexes was based on precipitation using an acid–base reaction. Precipitation is a process in which a solid forms from a solution when the concentration exceeds the solubility of the substance. In the case of MSZ, this process is strongly influenced by the ionic nature of MSZ. The ionized form of a drug is generally more soluble in water, while the non-ionized form is less soluble and may precipitate. MSZ has an amino group (-NH_2_) and a carboxylic acid group (-COOH) linked to a benzene ring. The presence of these functional groups favors the formation of anionic, zwitterionic, and cationic forms. In the cationic form, the amino group is protonated (-NH^3+^) and the carboxylic acid group remains uncharged (-COOH). In the anionic form, the carboxylic acid group loses a proton to become a carboxylate anion (-COO-), while the amino group remains uncharged (-NH_2_). In the zwitterionic form, mesalazine can carry both a positive charge on the amino group (-NH^3+^) and a negative charge on the carboxylate group (-COO-), resulting in an uncharged molecule on the surface [19].

**Figure 5:**
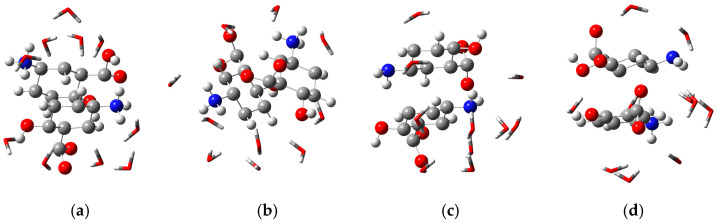
Interaction between mesalazine species and water molecules in a medium with the dielectric constant of water—3D. (**a**) Cationic; (**b**) zwitterionic; (**c**) anionic; (**d**) zwitterionic/anionic.

The formation of these precipitates was also investigated through Density Functional Theory (DFT) computational calculations. Two molecular structures of mesalazine were considered for each species at its respective pH value: cationic (pH 1.5), zwitterionic (pH 4.0), anionic (pH > 7), and a 1:1 mixture of zwitterionic and anionic forms corresponding to pH ~6.0. Each system was modeled in an interaction with ten explicit water molecules, and the dielectric constant of water was applied to simulate a solvated environment. This approach allowed us to evaluate the interactions between mesalazine and water in different protonation states. Figure 5 illustrates the spatial arrangement of these interactions.

The interaction energies between the species depicted in Figure 5 and water were obtained using Equation (1) [32]. The corresponding values, expressed in kcal·mol^−1^, are presented in Table 5. It can be observed that the interaction between the cationic species and water exhibits an energy of −1046.84 kcal·mol^−1^, indicating a strong interaction between this species and the solvent. This result also suggests that the cationic species has high solubility in water, and therefore its precipitation tendency is considerably lower. These findings are consistent with the results presented in Table 4.

Table 5 also presents the interaction energies of the zwitterionic, anionic, and mixed species (50% zwitterionic + 50% anionic). Notably, both the zwitterionic species and the mixture exhibited positive interaction energy values, indicating low solubility in water and, consequently, a higher tendency to precipitate. This observation is consistent with the data in Table 4, which shows approximately 95% precipitation for the species at pH 4 (zwitterionic) and around 75% precipitation for the species at pH 6 (50% zwitterionic + 50% anionic).

Computational modeling and experimental analysis assisted in the rational design of the methotrexate nanosuspension obtained using the bottom-up method. Computational studies determined the structural and electronic properties of isolated molecules and molecular clusters in order to evaluate the mechanism of methotrexate nanoparticle formation [29]. In previous studies, it was suggested that the MSZ concentration range that resulted in nanoscale particles was very limited, below 1.66 mg/mL MSZ [19]. However, in this study, it was demonstrated that controlling pH, agitation, and drip rate increased the MSZ concentration capable of forming nanoscale particles, exceeding 11 mg/mL. Therefore, studying the effect of pH is critical in the preparation of nanoparticles containing drugs with pH-dependent solubility, such as MSZ. At pH 1.5, approximately 56% of the drug precipitated; at a pH of 4.0, over 95% precipitated. They were zwitterionic when precipitated, and at a pH of 6.0, over 60% precipitated. At pH values below 1.5 or above 6.5, particle formation did not occur because the drug remained in the solution. Comparing the complexes formed at pH 6.0 with those at pH 1.5, the first presented volume and surface area (2457.61 Å^2^) were smaller than that of the complexes formed at a pH of 1.5, suggesting that the particles obtained at a pH of 6.0 are structurally more compact. The experimental approaches confirmed these results.

### 3.2. Effect of Incorporation of Chitosan in MSZ Nanoparticles

The pH values of 4.0 and 6.0 were selected for studies of CS incorporation due to their smaller particle sizes, higher precipitation percentages, and negative zeta potential values, making them suitable for incorporating positively charged polyelectrolytes such as chitosan. Conversely, negatively charged polyelectrolytes, such as hyaluronic acid, can interact via electrostatic attraction with particles obtained at a pH of 1.5, which exhibit a positive zeta potential.

CS incorporation into the delivery system can modify biological mechanisms, such as transport mechanisms, through biological membranes or mucus. Supramolecular interactions are involved in the formation of chitosan-based nanostructures. These non-covalent interactions are less energetic compared to covalent bonds, conferring many advantages. However, they present significant challenges in the development of engineered drug delivery systems through the manipulation of supramolecular interactions. Hence, a comprehensive understanding of the thermodynamic and mechanistic aspects involved will be important for obtaining more finely tuned systems [17,19,20].

**Figure 6:**
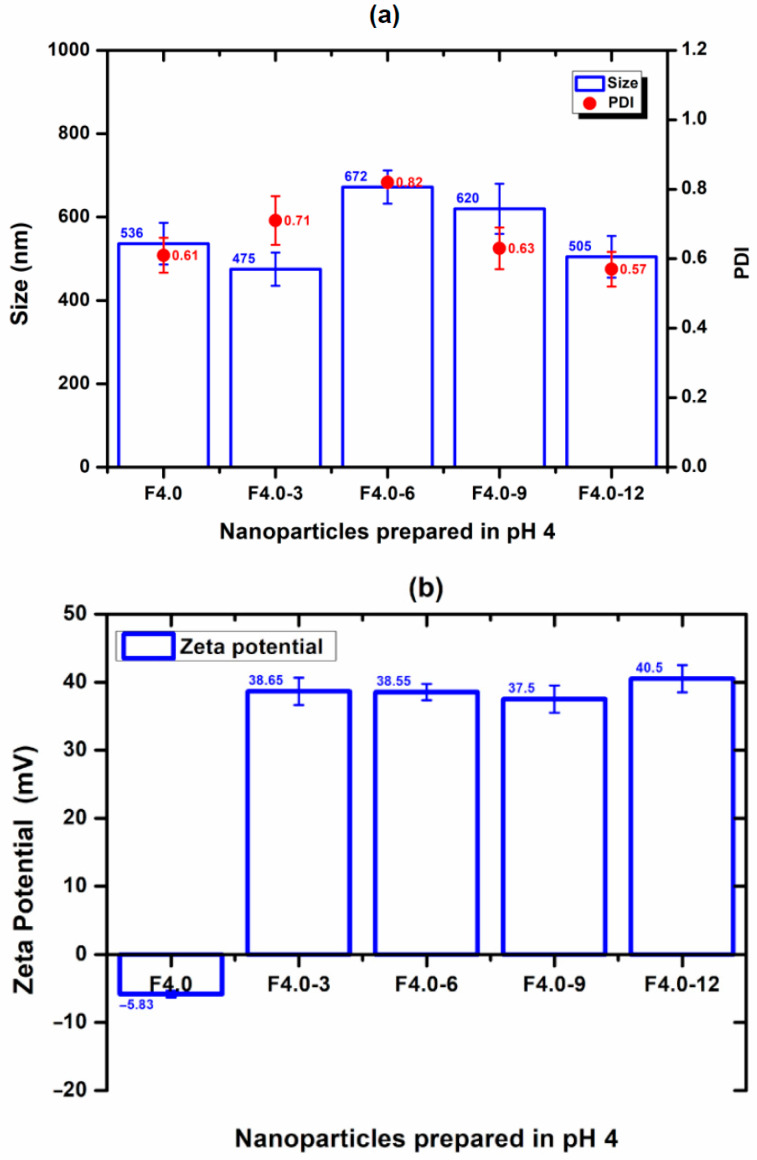
Effect of CS incorporation in MSZ-NPs prepared at a pH of 4.0: (**a**) Size (bar), PDI (circ), and (**b**) zeta potential (bar). Each point represents the mean ± SD of three experiments (*n* = 3). Nanoparticles without CS (F4.0), nanoparticles with 3% CS (F4.0-3), nanoparticles with 6% CS (F4.0-6), nanoparticles with 9% CS (F4.0-9), nanoparticles with 12% CS (F4.0-12).

**Figure 7:**
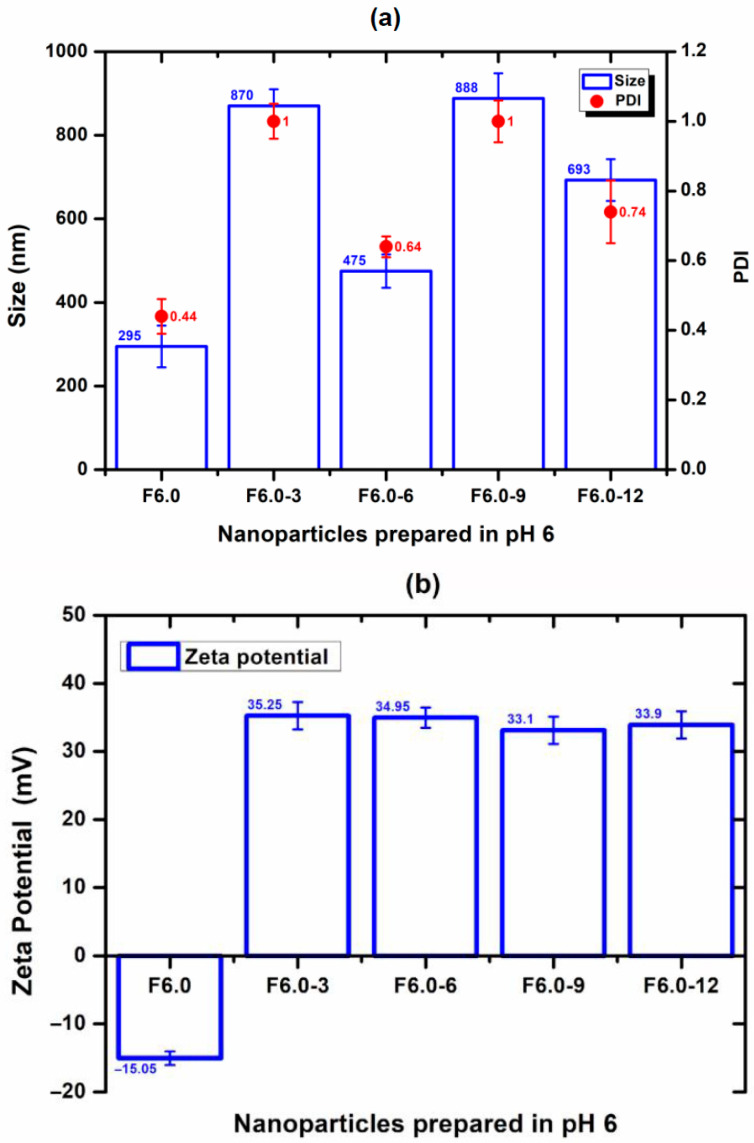
Effect of the incorporation process of CS on MSZ nanoparticles prepared at a pH of 6.0: (**a**) Size (bar), PDI (circ), and (**b**) zeta potential (bar). Each point represents the mean ± SD of three experiments (*n* = 3). Nanoparticles without CS (F6.0), nanoparticles with 3% CS (F6.0-3), nanoparticles with 6% CS (F6.0-6), nanoparticles with 9% CS (F6.0-9), nanoparticles with 12% CS (F6.0-12).

The nanoparticle prepared at a pH of 1.5 (F1.5) was excluded from this study due to its positive zeta potential, which does not favor interaction with CS and has a positive charge. For F4.0, nanoparticles prepared at a pH of 4.0, the effect of CS concentration on particle size, PDI, and zeta potential was evaluated and is shown in Figure 6. The incorporation of 3% to F4.0 and F4.0-3 resulted in the smallest particle size (Figure 6). The PDI values for the formulation with CS were like those observed with F4.0 (Figure 6). While F4.0 exhibited a negative zeta potential (−5.83 mV), the addition of CS promoted charge inversion, suggesting the adsorption of CS molecules on the surface of MSZ-NPs (Figure 6). The F6.0 formulation prepared at a pH of 6.0 exhibited a zeta potential of −15.05 mV. Upon CS incorporation, a charge inversion was also observed (Figure 7). The samples containing 6% of CS F6.0-6) exhibited the smallest particle size and the lowest PDI, suggesting that CS interacted with the particle surface in a more organized manner, leading to a more compact structure. The higher CS proportion required for F6.0 compared to F4.0 is likely due to the greater number of ionized carboxylic groups present on the F6.0 nanoparticles.

To design drug delivery systems with tailored properties, it is necessary to understand how the structural properties of particles, size, and charge can be controlled [14]. The particle synthesis process also influences the structural properties of particles. Thus, to evaluate the influence of the synthesis process, chitosan dispersions were added during the production of mesalazine nanoparticles prepared via acid–base precipitation. The sample obtained at a pH of 4.0 with 5% CS added during the MSZ-NP preparation process, F4.0-5, had a smaller size than F4.0 and F4.0-3. The zeta potential value of sample F4.0-5 was positive, but lower than the zeta potential value observed in sample F4.0-3. The addition of CS during the precipitation process at pH 4.0 may have enabled the formation of additional interaction points between the ionizable groups of MSZ and CS, and thereby promoted chain rearrangement, resulting in the formation of a more compact matrix and, consequently, a reduced particle size. Conversely, the zeta potential values of samples F6.0-6 and F6.0-5 were similar, indicating that the quantity of CS on the particle surface is comparable. A comparative analysis of the size values revealed that sample F6.0-5 exhibited the smallest size value compared to the other samples prepared at a pH of 6.0. Sample F6.0-5 showed improved features, such as size and PDI, and maintained adequate particle zeta potential values (Table 6). These are input parameters that must be considered when developing nanoparticles to adjust critical quality attributes such as size, polydispersity, and zeta potential, and they can influence the drug’s mucoadhesive properties.

### 3.3. Interaction of Mucin with F6.0 and F6.0-5

The interaction of nanoparticles with the biological interface is imperative to improve the efficacy of a drug, as it has the capacity to increase the amount of drug at the absorption or action site. Mucus, produced by cells of the intestinal epithelium, is composed of water, inorganic salts, lipids, enzymes, fatty acids, and glycoproteins. Mucin, the primary glycoprotein of mucus, is composed of branched and unbranched blocks. The interaction mechanism between the polymeric system and mucin involves supramolecular forces. This interaction occurs due to van der Waals forces, hydrogen bonds, and electrostatic and hydrophobic interactions [15,16,17,18,57,58].

**Figure 8:**
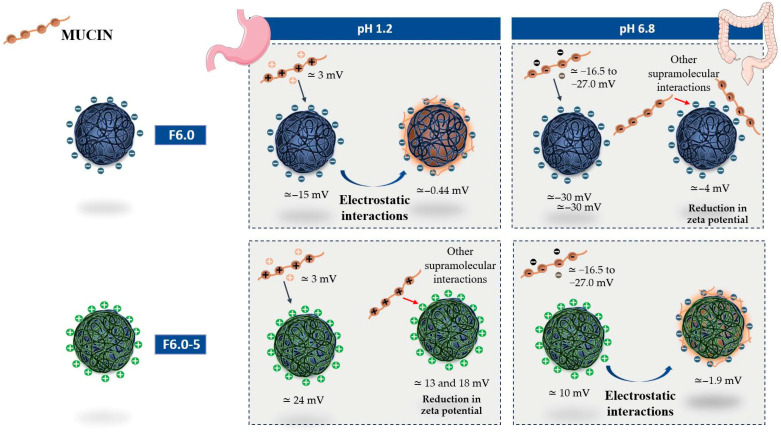
Schematic illustration of the interaction of mucin with MSZ nanoparticles, without chitosan (F6.0) and with CS (F6.0-5), at pH 1.2, simulating gastric pH, and at pH 6.8, simulating enteric pH, based on the results presented in Table 7.

In the present study, the interaction of mucin with MSZ-NP prepared at pH 6.0 were evaluated through variations in zeta potential values obtained at pH 1.2, simulating the gastric medium, and at pH 6.8, simulating the enteric medium. Figure 8 illustrates the results presented in Table 6. Mucin showed different zeta potential values at the tested pH. At pH 1.2, this protein showed positive zeta potential values close to 3 mV. At pH 6.8, the zeta potential values are dependent on the concentration of this protein, varying from −16.5 mV for a concentration of 100 µg/mL to −27 mV at the highest concentration tested, 300 µg/mL. On the other hand, the zeta potential values of the tested nanoparticles varied with the pH. The ZP values of the F6.0 nanoparticle ranged from −14.6 at pH 1.2 to −29.6 at pH 6.8. The F6.0-5 nanoparticle showed positive values at both pH levels, being lower at pH 6.8. It was observed that both nanoparticles (F6.0 and F6.0-5) interacted with mucin at a pH that simulates gastric pH, pH 1.2. However, it is hypothesized that these interactions are governed by different supramolecular interactions. The interaction of mucin with the F6.0 formulation, consisting of nanoparticles without CS, resulted in an electrostatic interaction, leading to an increase in the ZP value relative to the ZP of the F6.0 nanoparticle. The interaction of mucin with F6.0-5, which exhibits positive zeta potential values at this pH, showed a reduction in the zeta potential value, which may indicate that other supramolecular interactions, such as hydrophobic bonds and Van der Waals forces, are involved. At pH 6.8, the nanoparticles exhibit inverse behavior when incubated with mucin. The formulation F6.0 possibly interacts with mucin through supramolecular interactions other than electrostatic interaction, while at F6.0-5, electrostatic interaction is possibly favored.

**Table 7:** Effect on zeta potential values of incubating mucin with MSZ-NPs with or without CS in 0.1 N HCl (pH = 1.2) and phosphate-buffered medium (pH = 6.8).

	Mucin Concentration (µg/mL)	Zeta Potential (mV)	
		Gastric Medium (HCl 0.1 N— pH 1.20)	Intestinal Medium (Phosphate-Buffered Medium, pH 6.8)
Mucin	100	2.3	−16.5
	200	2.7	−19
	300	3.0	−27
F6.0	-	−14.6	−29.6
	100	0.205	−6.56
	200	−0.307	−3.99
	300	−0.443	−4.27
F6.0-5	-	26.8	10.2
	100	16.53	3.75
	200	13.37	−0.81
	300	18.47	−1.9

Mucoadhesion is a highly complex mechanism influenced by various physicochemical and biological factors that affect the interaction between the polymer and the mucus, influencing the effectiveness of mucoadhesive systems in pharmaceutical applications. Among the different factors, the positive electrical charge of the polymer tends to interact with the mucin, which has a negative charge, depending on the pH of the medium, through electrostatic interactions [16,17,18,58]. Polymers with functional groups such as hydroxyls, carboxyls, and amines can form hydrogen bonds with mucin, strengthening adhesion. Polymers with higher molecular weight generally exhibit greater adhesion strength due to the greater number of contact points with the mucus. Polymers with more flexible chains can increase the contact area with mucus, enhancing the polymer’s mucoadhesive properties. The pH of the medium can alter the ionization of the polymer’s and mucin’s functional groups, affecting the strength of the interactions. The strategy for modulating the system to interact with mucus is complex and should incorporate a better understanding of nano-biointerfacial interactions to achieve the desired biological response.

## 4. Conclusions

In recent years, nanotechnology has experienced significant advances to be made in pharmaceutical research, particularly in the treatment of complex diseases such as cancer, inflammatory diseases, and parasitic diseases. Nanostructured systems, in which drugs are compartmentalized in restricted environments, alter the various characteristics and properties of drugs, differentiating them from traditional formulations. When a drug is compartmentalized in a nanostructured system, pharmacokinetic parameters such as absorption, distribution, and elimination are modulated not only by the properties of the drug itself but also by the characteristics of the nanocarriers [11,12,13,14,15,16,17,18,19].

The rational design of drug delivery systems is based on the manipulation of components and processes with the aim of achieving the desired properties. The selection of system components is critical and must be guided by the desired biological response. The definition of the biological target is fundamental for selecting the qualitative aspects of particle composition. However, to achieve the outlined objectives, it is essential to understand the mechanisms governing particle formation.

Multiple parameters can impact particle formation, including the type of polymer, the pH of the synthesis medium, and the ratio of materials used. These factors can influence the attractive forces between the polymer and drugs. Therefore, a comprehensive understanding of the factors involved in particle formation and preparation, as well as their effects on the structural properties of nanostructured systems, is essential to the development of drug delivery systems with tailored properties for desired biological performance.

This study proposed a set of guiding considerations for the rational design of nanostructured systems with mucoadhesive properties for the delivery of drugs with pH-dependent solubility, such as MSZ. Chitosan, which has a positive charge over a wide pH range, was the selected polymer. Conversely, MSZ particle formation is strongly influenced by pH, thus modulating the ionic form and, consequently, their supramolecular interactions. Molecular modeling showed that cationic MSZ exhibits negative interaction energies, suggesting the formation of more stable complexes, while anionic species form fewer stable complexes. At a pH of 1.5, larger, positively charged particles are produced, while at pHs of 6.0 and 4.0, smaller particles with a negative charge are produced. These results suggest that ionic forms, which are modulated by pH, govern the equilibrium of supramolecular interactions and determine particle size, charge, and stability. In this work, particles obtained at pHs of 4.0 and 6.0 were selected for CS incorporation due to their favorable properties: nanoscale particles and negative zeta potential. Using computational modeling and experimental approaches, it was possible to establish conditions for preparing nanoparticles with a high concentration of MSZ, which is very important for this drug.

## Figures and Tables

**Table 1:** Composition of MSZ particles.

	F1.5	F4.0	F6.0
MSZ (mg)	200	200	200
HCl 0.5 M (mL)	5	5	5
H_2_O (mL)	10	10	10
NaOH 1 M (mL)	1.00	2.48	3.18
Initial pH	1.2	1.2	1.2
Final pH	1.5	4.0	6.0
Final MSZ (mg/mL)	12.5	11.44	11.00

**Table 2:** Interaction energy of mesalazine molecules in different ionic states: cationic, zwitterionic, and anionic.

Number of Mesalazine Molecules	Interaction Energy (kcal·mol^−1^)		
	Cationic	Zwitterionic	Anionic
2	−9.35	−9.22	−8.73
5	−40.30	−38.42	−37.02
10	−92.75	−94.30	−88.72
15	−148.22	−145.54	−140.75
20	−210.88	−196.50	−193.98
25	−263.20	−257.72	−253.49
30	−327.91	−325.52	−313.92

**Table 3:** Volume and superficial area of MSZ clusters in anionic, zwitterionic, and cationic charge states.

MSZ Molecules’ Number	Cluster Volume (A^3^)			Clusters’ Superficial Area (A^2^)		
	Cationic	Zwitterionic	Anionic	Cationic	Zwitterionic	Anionic
2	297.56	291.05	286.14	268.26	259.12	257.09
15	2456.99	2417.00	2347.77	1404.54	1348.82	1389.75
18	2963.43	2890.42	2872.43	1667.65	1594.71	1553.19
20	3300.18	3219.04	3163.85	1850.17	1827.18	1766.48
22	3654.87	3576.35	3495.37	1995.08	1896.45	1906.65
24	4002.79	3878.67	3863.15	2191.81	2121.81	2027.50
28	4659.50	4559.63	4494.84	2511.73	2427.85	2236.77
30	5008.32	4889.54	4816.85	2666.82	2463.60	2457.61

**Table 4:** Particle size, polydispersity index (PDI), zeta potential, and MSZ precipitated. Each point represents the mean ± SD of three experiments.

pH	Size (nm)	PDI	Zeta Potential (mV)	MSZ Precipitated (%)
1.5	936.7 ± 20.2	0.588 ± 0.50	+8.5 ± 0.2	51.6 ± 5.5
4.0	556.2 ± 10.4	0.478 ± 0.60	−22.3 ± 1.4	95.1 ± 2.4
6.0	145.9 ± 41.6	0.497 ± 0.22	−31.6 ± 0.9	75.5 ± 2.4

**Table 5:** Interaction energy of mesalazine species with water.

System	Interaction Energy (kcal·mol^−1^)			
Mesalazine + 10 Water molecules	Cationic	Zwitterionic	Anionic	50% Zwitterionic/50% Anionic
	−1046.84	8.80	−6.59	4.25

**Table 6:** Effect of the process variable of CS incorporation into MSZ nanoparticles on particle size and zeta potential values. Each point represents the mean ± SD of three experiments (*n* = 3).

Samples	Size (nm)	Zeta Potential (mV)
F4.0	442.4 ± 50	−5.8 ± 0.5
F4.0-3	475.3 ± 40	38.6 ± 1.2
F4.0-5	327.4 ± 5.8	24,7 ± 1,7
F6.0	264.8 ± 44	15.0 ± 1.0
F6.0-6	475.0 ± 64	34.9 ± 1.0
F6.0-5	169.3 ± 9.7	31.8 ± 2.0

## Data Availability

The raw data supporting the conclusions of this article will be made available by the authors on request.

## Abbreviations

The following abbreviations are used in this manuscript:

MSZ	Mesalazine
MSZ-NP	Mesalazine nanoparticles
CS	Chitosan
